# Supplementary material for: Longitudinal Follow-Up Using the Heel Enthesitis Magnetic Resonance Imaging Scoring System (HEMRIS) Shows Minimal Changes in Heel Enthesitis Assessed in Spondyloarthritis and Psoriasis Patients
Source: J Pers Med. 2022 Oct 25;12(11):1765. doi: 10.3390/jpm12111765 (PMC9693157; doi:10.3390/jpm12111765)

Supplemental Table S1. Total HEMRIS scores and change in total HEMRIS scores at Achilles tendon, plantar fascia and Achilles tendon and plantar fascia combined.

	Start of one-year interval	End of one-year interval	Change scores	p-value*
**Achilles tendon (summated scores left + right ankle)**				
Total MRI-scores, n=	72	67	67	
Total HEMRIS entheseal inflammation score (0-24), median (IQR)	1.0 (0.0-2.0)	0.0 (0.0-1.0)	0.0 (0.0-0.0)	0.28
Total HEMRIS structural damage score (0-24), median (IQR)	1.0 (0.0-2.0)	1.0 (0.0-2.0)	0.0 (0.0-0.0)	0.32
Total missing MRIs, reasons:				
Missing, patient lost to follow-up (n=)	2	6	6	
Missing, MRI quality (n=)	2	3	3	
**Plantar fascia (summated scores left + right ankle)**				
Total MRI-scores, n=	72	68	68	
Total HEMRIS entheseal inflammation score (0-18), median (IQR)	0.0 (0.0-0.8)	0.0 (0.0-0.0)	0.0 (0.0-0.0)	0.31
Total HEMRIS structural damage score (0-24), median (IQR)	0.0 (0.0-1.8)	0.0 (0.0-2.0)	0.0 (0.0-0.0)	0.68
Total missing MRIs, reasons:				
Missing, lost to follow-up (n=)	2	6	6	
Missing, MRI quality (n=)	2	2	2	
**Total (Achilles tendon + plantar fascia, summated scores left + right ankle)**				
Total MRI-scores, n=	72	68	67	
Total HEMRIS entheseal inflammation score (0-42), median (IQR)	1.0 (0.0-3.0)	1.0 (0.0-2.0)	0 (-1.0 - 0.0)	0.14
Total HEMRIS structural damage score (0-48), median (IQR)	1.0 (0.0-3.8)	1.0 (0.0-4.0)	0.0 (0.0-0.0)	0.89
Total missing MRIs, reasons:				
Missing, patient lost to follow-up (n=)	2	6	6	
Missing, MRI quality (n=)	2	3	3	

Table Legend. *= Wilcoxon Signed Rank Test, n=total number of MRI-scans, HEMRIS = Heel Enthesitis MRI Scoring System, IQR = interquartile range, MRI = magnetic resonance imaging

Supplemental Table S2: Patients' characteristics at follow-up

Patients' characteristics at 52 and 104 weeks (pooled data). Patients that were lost to follow-up were excluded from the analysis.

	Disease category (at time of inclusion)			
	Psoriasis	Psoriatic arthritis	Ankylosing spondylitis	All
Total one year intervals, N =	23	23	24	70
General disease activity:				
Pso: moderate-severe psoriasis, n (%)	2 (8.7)	NA	NA	NA
PsA: MDA, n (%)	NA	14 (60.9)	NA	NA
Missing, n (%)	NA	3 (13.0)	NA	NA
AS: BASDAI score ≥ 4, n (%)	NA	NA	21 (87.5)	NA
Missing, n (%)	NA	NA	1 (4.2)	NA
Medication:				
Current DMARD use, n (%):	1 (4.3)	8 (34.8)	2 (8.3)	11 (16.2)
Missing, n (%)	0	1 (4.3)	1 (4.2)	2 (2.9)
Current NSAID use, n (%):	2 (8.7)	5 (21.7)	15 (62.5)	22 (32.4)
Missing, n (%)	0	1 (4.3)	1 (4.2)	2 (2.9)
Inflammatory markers:				
ESR, median (IQR):	5.0 (2.0 - 13.0)	3.0 (2.0 - 6.0)	5.0 (2.0 - 9.3)	4.5 (2.0 -7.0)
Missing, n (%)	2 (2.9)	0	0	2 (2.9)
CRP, median (IQR):	1.6 (0.5 - 9.2)	3.5 (2.2-6.2)	1.3 (0.7 - 3.9)	2.2 (0.9-4.9)
Missing, n (%)	1	0	1	2 (2.9)
Total				
Local disease activity at the enthesis:				
Achilles tendon, N enthesis =	46	46	48	140
Clinical enthesitis, n (%)	4 (8.7)	0	4 (8.3)	8 (5.7)
Missing, n (%)	0	2 (4.3)	8(16.7)	10 (7.1)
Plantar fascia, N enthesis =	46	46	48	140
Clinical enthesitis, n (%)	6 (13.0)	5 (10.9)	0	11 (7.9)
Missing, n (%)	0	2 (4.3)	0	10 (7.1)

Table legend. Abbreviations: AS = ankylosing spondylitis, BASDAI = Bath Ankylosing Spondylitis Disease Activity Index, CRP = C-reactive protein, DMARD = disease-modifying anti-rheumatic drugs, ESR = erythrocyte sedimentation rate, IQR = interquartile range, Pso = psoriasis, PsA = psoriatic arthritis, MDA = minimal disease activity, NA = not applicable, NSAID = non-steroidal anti-inflammatory drugs.

Supplemental Table S3. Change in HEMRIS subscores after longitudinal follow-up

Table legend.

	Achilles tendon	Plantar fascia
Total observations, n=	137	138
Tendon thickening		
- Increase, n(%)	1 (0.7)	1 (0.7)
- Decrease, n(%)	0 (0)	3 (2.2)
Tendon hypersignal T1W:		
- Increase, n(%)	1 (0.7)	1 (0.7)
- Decrease, n(%)	5 (3.6)	3 (2.2)
Bone spur:		
- Increase, n(%)	1 (0.7)	0 (0)
- Decrease, n(%)	0	1 (0.7)
Tendon erosion:		
- Increase, n(%)	0 (0)	0 (0)
- Decrease, n(%)	0 (0)	0 (0)
Bone marrow edema:		
- Increase, n(%)	0 (0)	2 (1.4)
- Decrease, n(%)	0 (0)	1 (0.7)
Tendon hypersignal T2W:		
- Increase, n(%)	0 (0)	2 (1.4)
- Decrease, n(%)	6 (4.4)	3 (2.2)
Peritendon hypersignal:		
- Increase, n(%)	3 (1.8)	5 (3.6)
- Decrease, n(%)	10 (7.3)	11 (28.9)
Retrocalcaneal bursitis:		
- Increase, n(%)	10 (7.3)	NA
- Decrease, n(%)	10 (7.3)	NA

Table legend. Abbreviations: NA = not applicable, T1W = T1-weighted images, T2W = T2-weighted images

Supplemental Figure S1: Flowchart

Clinical evaluations at each time point (baseline, one year follow up and two years follow-up).

Figure legend. Abbreviations: AS = ankylosing spondylitis, FU = follow-up, Pso= psoriasis, PsA = psoriatic arthritis, MRI= magnetic resonance imaging

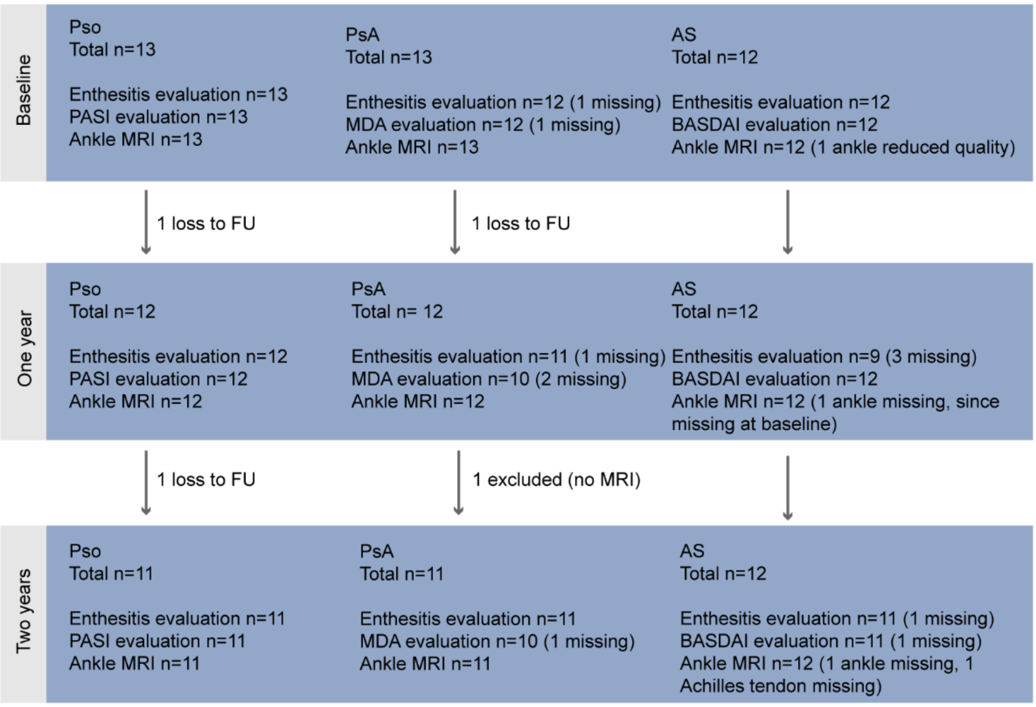

Supplemental Figure S2: Change in the HEMRIS in Pso patients with and without progression to PsA

No differences were observed in respective HEMRIS inflammation and structural damage results at the Achilles tendon (A and B), plantar fascia (C en D) and plantar fascia and Achilles tendon and plantar fascia combined (E and F) at inclusion in Pso patients that later developed PsA (‘converter’, n=2), in comparison with Pso patients that did not developed PsA. (‘no converter’, n=11).
Figure legend. Abbreviations: Pso = psoriasis, PsA = psoriatic arthritis.

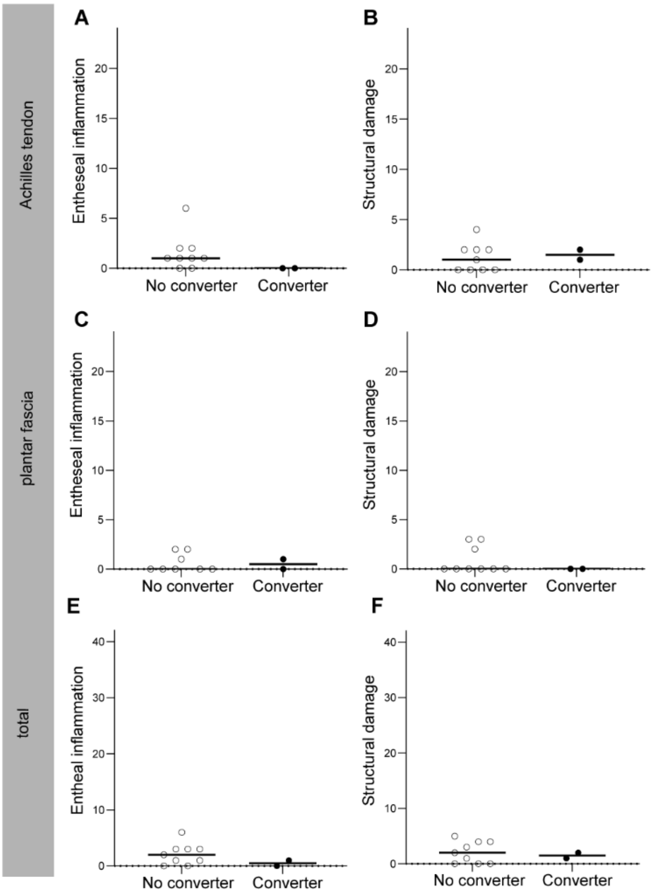

Supplement: Supplementary file 1 [file jpm-12-01765-s001.zip › jpm-1955343-supplementary.pdf]
